# Defining the Pre-exposure Prophylaxis Care Continuum Among Recently Incarcerated Men at High Risk for HIV Infection: Protocol for a Prospective Cohort Study

**DOI:** 10.2196/31928

**Published:** 2022-02-10

**Authors:** Matthew Murphy, Collette Sosnowy, Brooke Rogers, Siena Napoleon, Drew Galipeau, Ty Scott, Jun Tao, Justin Berk, Jennifer Clarke, Amy Nunn, Philip A Chan

**Affiliations:** 1 Brown University Providence, RI United States; 2 The Miriam Hospital Providence, RI United States

**Keywords:** HIV, PrEP, criminal justice system, incarceration, criminal justice, pre-exposure prophylaxis, prison system

## Abstract

**Background:**

HIV disproportionately impacts criminal justice–involved individuals, including men who experience incarceration. Men make up the vast majority of those experiencing incarceration as well as those newly diagnosed with HIV infection. Pre-exposure prophylaxis (PrEP) is a highly effective biomedical intervention that significantly reduces the risk of HIV acquisition. However, implementation in criminal justice systems is limited. Little is known about effective PrEP implementation and use in this unique public health context.

**Objective:**

The aim of this study is to characterize the experience of implementing PrEP clinical care in a criminal justice setting for men vulnerable to HIV acquisition.

**Methods:**

This article describes a PrEP care continuum for men experiencing incarceration who are at increased risk of HIV acquisition, which can help conceptualize approaches to evaluating PrEP implementation.

**Results:**

The outlined study will enroll 100 men experiencing incarceration at high risk for HIV acquisition prior to release into the community. The goal is to initiate PrEP prior to release and link individuals to PrEP providers in the community, capturing barriers and facilitators to PrEP use during this uniquely vulnerable time period for HIV acquisition.

**Conclusions:**

Based on the proposed care continuum and what is known about HIV risk and prevention efforts in the criminal justice context, we outline key future research efforts to better understand effective approaches to preventing HIV infection among this vulnerable population. The described approach presents a powerful public health opportunity to help end the HIV epidemic.

**International Registered Report Identifier (IRRID):**

DERR1-10.2196/31928

## Introduction

There are approximately 38,000 new HIV diagnoses in the United States annually [1]. Men make up the vast majority of new diagnoses, accounting for over 80% of these cases [1]. The most frequent mechanisms of HIV acquisition are sexual contact and injection drug use [1]. Men who have sex with men (MSM), including men who participate in transactional sex with other men, have a high burden of HIV and comprise approximately 70% of all HIV diagnoses [1,2]. There are also significant racial disparities within the United States among incident cases of HIV annually, with racial and ethnic minorities comprising a disproportionate number of new cases compared to White individuals. Black/African American men account for 39% of new HIV diagnoses, yet represent only 18% of the general male population, and Hispanic/Latino men accounted for 29% of new HIV diagnoses while only making up 13% of the general population [3]. Among MSM, Whites have a 1 in 11 lifetime risk of HIV acquisition, compared to Black/African American men, who have a 1 in 2 risk and Hispanic/Latino MSM, who have a 1 in 5 lifetime risk [4]. The most frequent mechanisms of HIV acquisition are sexual contact (88%) and injection drug use (7%-11%) [1].

Criminal justice–involved individuals in the United States are among the most vulnerable to and heavily impacted by HIV [5]. Individuals with a history of incarceration have a rate of HIV infection that is 3-5 times that of their nonincarcerated counterparts [6]. One in 7 people living with HIV pass through the criminal justice system each year [7]. The rate of incarceration for men is 10 times greater than that for women [8]. Black/African American men are also 6 times as likely and Hispanic/Latino men are more than twice as likely as White men to be incarcerated [9]. Criminal justice involvement is highly prevalent among people who inject drugs, with an estimated 72.2% reporting a history of incarceration [5]. Following release, criminal justice–involved individuals are more likely to participate in sexual and substance use behaviors, including injection drug use, that place them at increased risk of HIV infection and overdose [10-17]. Complex, intersecting social and structural forces place racial and ethnic minority populations in particular at increased vulnerability to both criminal justice involvement and HIV acquisition.

Although incarceration represents an opportunity to test criminal justice–involved individuals who are at risk for HIV and link them to HIV and/or pre-exposure prophylaxis (PrEP) care in the community as needed, surveys conducted in criminal justice settings show uneven uptake of HIV testing, preventive, and treatment services among criminal justice–involved individuals [18]. PrEP is currently available as an oral medication in two forms: tenofovir disoproxil fumarate and emtricitabine (TDF/FTC) and tenofovir alafenamide and emtricitabine (TAF/FTC). Both medications are highly effective at preventing HIV transmission among MSM [19,20]. TDF/FTC has also been shown to be effective at preventing HIV transmission through receptive vaginal intercourse [21] as well as injection drug use [22]. Importantly, research has shown different concentrations of TDF/FTC in vaginal compared to anorectal tissue, leading to different approaches to initiate PrEP and measure adherence among men and women [23].

Despite its effectiveness, PrEP uptake in real-world settings among multiply marginalized populations, including criminal justice–involved individuals, remains low [24]. Although some research has demonstrated PrEP interest among criminal justice–involved individuals [25,26], little is known about the barriers to PrEP implementation during incarceration or the period immediately postrelease from incarceration [27,28]. What is known is that there are expressed reasons to decline PrEP initiation unique to the correctional setting such as institutional mistrust and the high degree of uncertainty in the postrelease period [29,30]. A PrEP care continuum is a useful conceptual tool for identifying facilitators and barriers to PrEP use, adherence, retention, and persistence in PrEP care [31]. In this article, we propose a modified PrEP care continuum for criminal justice–involved populations and then describe an approach to implementing and assessing PrEP care among criminal justice–involved men at high risk of HIV infection.

## Methods

### Defining the PrEP Care Continuum for Criminal Justice–Involved Populations

**Figure 1:**
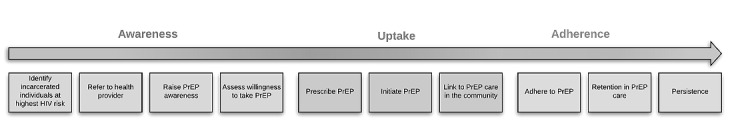
PrEP care continuum for incarcerated populations. PrEP: pre-exposure prophylaxis.

The PrEP care continuum can help to conceptualize and structure PrEP programming in the criminal justice system, including PrEP awareness, uptake, and adherence, and retention in PrEP care [31]. The PrEP care continuum has been used as a tool to better understand the barriers and facilitators to PrEP care for other vulnerable populations [32-34], but it has yet to be applied to or extensively studied in the criminal justice–involved population [35]. Adapting a PrEP continuum unique to criminal justice environments may help clinical providers and policy makers to identify gaps and barriers to uptake and develop specific programming to address them. Ideally, these efforts will enable successful identification of individuals who meet the clinical criteria for PrEP use, as well as frame the development of effective interventions to increase PrEP uptake, adherence, and retention in care, particularly during the postrelease period (Figure 1).

There are a number of elements to consider for the successful implementation of its use within the criminal justice setting. Although many individuals who pass through the criminal justice system are at increased risk for HIV, the Centers for Disease Control and Prevention (CDC) provides guidance for the clinical criteria for PrEP use that should be used when identifying individuals that are indicated for PrEP [36]. Additionally, for PrEP to be effective, adherence to the medication is crucial [22,37]. Finally, persistence in PrEP care is defined as maintaining all aspects of recommended PrEP clinical care during a defined period (ie, attending care appointments, attending lab visits, taking medication as prescribed) [38]. For individuals with ongoing HIV risk, persistence in PrEP care should extend for as long as it is indicated.

Within the three phases of the continuum (PrEP awareness, uptake, and adherence), the steps are the following: (1) identify incarcerated individuals at highest risk for contracting HIV, (2) refer to health care provider within the criminal justice system, (3) increase PrEP awareness among those individuals, (4) assess willingness to take PrEP, (5) prescribe, and (6) initiate PrEP while still incarcerated, (7) link to PrEP care in the community, (8) support adherence to PrEP, (9) retain individuals in care, and (10) support persistence in PrEP care. This multistep process highlights the potential complexity of implementing a clinical intervention within a criminal justice context and then linking individuals to clinical care in the community upon release.

The challenges of successfully defining and implementing a care continuum have been more thoroughly studied among individuals who are living with HIV. Interventions to diagnose, initiate treatment for, and link people living with HIV to care in the community have become an important public health priority with a significant impact on the trajectory of the epidemic in the United States [5,39]. In order to broaden the public health impact of using the criminal justice system as a potential focal point for effective HIV prevention interventions, it is important to understand effective implementation strategies and interventions to promote PrEP uptake among the diverse populations that are both disproportionately impacted by HIV and criminal justice involvement. 

### The PrEP Care Continuum

#### Identify Incarcerated Individuals at Highest Risk for Contracting HIV

The variability in the availability of HIV screening and preventive services in the correctional setting presents one of the biggest challenges to successfully implementing PrEP care in the criminal justice system. This challenge is compounded in part by the potentially high volume of criminal justice–involved individuals who meet CDC criteria for PrEP initiation, but who may not self-identify as being at risk for HIV. There may be a number of reasons why individuals do not self-identify as being at risk for HIV acquisition including stigma, fear of legal repercussions for disclosing criminal behavior, or a lack of awareness of the risk of acquiring HIV from certain behaviors [40-42]. Perhaps the greatest public health benefit of PrEP use would be among pretrial individuals who are detained in the country’s jail systems [43]. With a high volume of individuals who often spend a short period of time in confinement, quickly identifying individuals who are at increased risk of HIV acquisition and then linking them to HIV preventive care in the community poses significant challenges but could have a significant public health benefit. A different approach for identifying individuals who would benefit from HIV preventive care among the sentenced population, many of whom spend months or years incarcerated, warrants tailored clinical tools that acknowledge unique HIV acquisition risks not currently addressed by CDC guidelines, which focus on risk behaviors during the prior 6 months [43]. For individuals incarcerated for greater than 6 months, their risk for HIV acquisition is unlikely to be captured using this criterion, particularly as individuals prepare for reentry into the community postincarceration [18].

#### Refer to Health Care Provider Within the Criminal Justice System

For individuals at an increased risk of HIV acquisition, referral to a health care provider in the criminal justice setting who could order HIV, sexually transmitted infection, and other lab testing necessary to initiate PrEP is an important and often limiting step. Given the variability in access to medical providers in the criminal justice setting, particularly providers who are knowledgeable about PrEP, this may present a unique challenge to PrEP care implementation [44]. The identification and training of health care providers who could assess for PrEP eligibility and initiate the clinical care associated with its use will be important in this setting [43,44].

#### Increase PrEP Awareness Among Those Individuals

The little that is known about PrEP awareness in the criminal justice system has demonstrated a lack of knowledge but significant interest [24-26]. Encounters with health care providers and public health support staff in the criminal justice setting can serve as important opportunities to discuss the benefits of PrEP use and improve an individual’s understanding of their own risk of HIV acquisition.

#### Assess Interest in, Willingness to, and Barriers to Taking PrEP

Although there is interest among incarcerated individuals in taking PrEP while in the criminal justice setting [45], it is important to characterize PrEP initiation patterns, hesitancy related to its use, and the influence of barriers both within the criminal justice system and upon reentry into the community. Components of this element of the PrEP care continuum are largely unknown and are likely to vary given the diverse makeup of those who experience incarceration, their socioeconomic context in the community, and the behaviors that put individuals at increased risk of HIV acquisition.

#### Prescribe PrEP

Many criminal justice–involved individuals experience barriers to accessing primary and preventive clinical care in the community [46]. Therefore, completing the clinical assessments and laboratory testing necessary to initiate PrEP during a period of detention provides significant advantages in promoting PrEP use. Ensuring individuals are HIV-negative, evaluating individuals for renal dysfunction, and assessing them for hepatitis B infection—disease processes that all disproportionately impact the criminal justice–involved population—facilitates identification of the few potential clinical limitations for PrEP use [47,48]. In addition to limited accessibility of PrEP providers and the clinical assessment needed to safely initiate PrEP, cost of the medication to criminal justice institutions may pose a barrier to its implementation. Onsite availability and prompt dispensation, particularly for pretrial individuals who may be spending only a brief period in detention, may pose a logistical challenge to criminal justice institutions and their clinical service providers [43].

#### Initiate PrEP Care While Still Incarcerated

There is a well-documented risk of HIV infection among criminal justice–involved individuals during the period immediately following release [46]. Ideally, criminal justice–involved individuals at increased risk for HIV acquisition would initiate PrEP prior to release. This strategy would also allow for observation and evaluation of side effects, while providing an opportunity to initiate a protective biomedical intervention that can be continued in the community. Providing individuals with medication upon release may improve adherence in the crucial postrelease period, while reducing a potential barrier to HIV preventive care in this potentially chaotic period [29,37].

#### Link to PrEP Care in the Community

Linkage to care in the community poses another significant challenge to PrEP care among criminal justice–involved individuals. Currently, providing continuity of care for both pretrial individuals as well as those sentenced to a period of confinement poses a challenge, particularly for those who are HIV-positive, are impacted by substance use disorder, or have general medical needs [49,50]. The availability and accessibility of PrEP care providers is likely to vary significantly based on geographic region, an individual’s insurance status, and the existence of other health linkage programs unrelated to PrEP care [51,52].

#### Support Adherence to PrEP

Although providing incarcerated individuals with a supply of medication upon release is likely to improve adherence by reducing a crucial barrier to PrEP access, adherence during the postrelease period is largely unknown and unstudied [43,53]. Given the importance of adherence to PrEP’s effectiveness, adherence patterns during the postrelease period represent an important knowledge gap and one that is likely to require tailored interventions to improve. This study uses both biological markers of PrEP adherence through dried blood spot measurements of TDF/FTC concentrations as well as self-reported measures of PrEP adherence.

#### Support Retention and Persistence in PrEP Care

Retention and persistence in PrEP care pose a number of challenges in “real-world” community settings [31]. It is likely, although largely unknown, that retention and persistence in PrEP care will also pose a significant albeit unique challenge to those with criminal justice involvement given the barriers that have been well documented in retaining individuals with criminal justice involvement in HIV care [31], hepatitis C care, general primary care, and preventive care, to name a few [54]. By establishing a prospective cohort of individuals returning to the community postincarceration, the outlined methodological approach will allow for the monitoring of changes in HIV acquisition risk behaviors as well as indications for continued PrEP use.

### Ethics Approval

The study underwent review and approval by the Lifespan Institutional Review Board's special committee on research conducted among incarcerated populations. Additional review and approval was provided by the Rhode Island Department of Corrections' Ethical Review Advisory Group.

## Results

**Figure 2:**
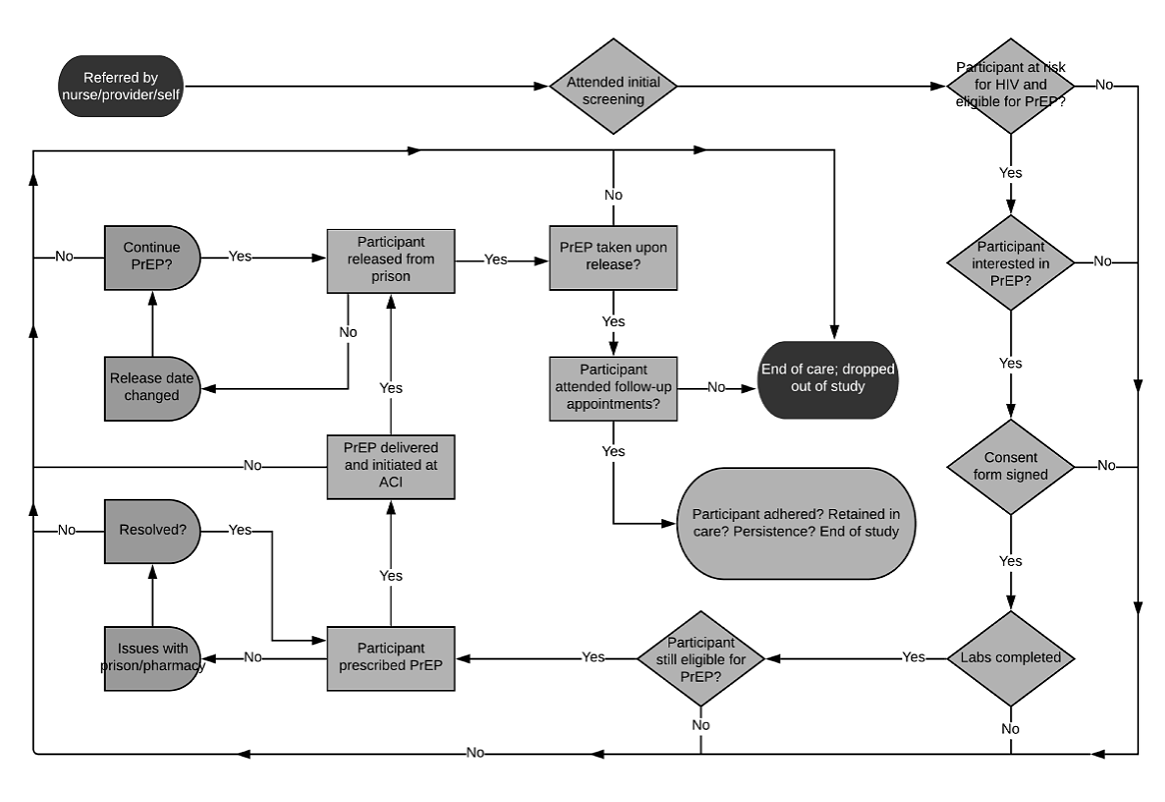
Implementing PrEP care continuum for criminal justice–involved populations. ACI: adult correctional institutions; PrEP: pre-exposure prophylaxis.

Given that men make up more than 90% of individuals who are incarcerated, and also comprise a majority of new HIV cases, we have proposed a study that focuses on PrEP uptake among men currently experiencing incarceration. This includes all men experiencing incarceration, including those detained in pretrial facilities and those sentenced to a period of confinement. The study is an open, prospective cohort study of 100 men who are currently incarcerated at the Rhode Island Department of Corrections (RIDOC) and who are scheduled to be released within one month of being enrolled in the study. RIDOC is a unique study environment, as it is a unified correctional system with all statewide pretrial and sentenced individuals detained on one campus under a single administrative structure. Every individual processed at RIDOC undergoes a nurse-led medical evaluation at intake (Figure 2). Individuals who report behaviors that would place them at increased HIV risk (condomless sex with multiple sexual partners; sex work and/or transactional sex; or injection drug use) are then referred to a general medical provider for potential study enrollment and/or initiation of PrEP clinical care through standard medical screening procedures. The existing nurse evaluation is brief and not intended to be a comprehensive evaluation of HIV acquisition risk. Other staff and medical providers can also refer individuals to the PrEP provider and program if they determine an individual to be at increased risk of HIV acquisition.

During the meeting with the PrEP clinical provider, the provider and patient discuss HIV risk, the provider assesses the patient’s baseline knowledge and interest in using PrEP, and the provider requisitions the necessary lab work and obtains the patient’s medical history to determine whether the patient is a candidate for PrEP use per CDC guidelines. For those interested in participating in the study, written informed consent is obtained and clinical documentation is shared with the research team at a community partner site. A PrEP navigator then subsequently meets with the patient and helps to coordinate postrelease PrEP care including measures of adherence and ongoing HIV acquisition risk. PrEP clinical care and enrollment in this study are centered largely on the pretrial population housed in RIDOC’s intake facility, which saw approximately 13,000 commitments during 2019 [55].

Once an interested individual is clinically screened and determined to be eligible for PrEP, TDF/FTC is prescribed, and the medication is delivered to the detention facility so that the individual may begin taking the medication prior to being released into the community; they are provided with a short-term supply upon release. Individuals are referred to a PrEP clinic in the community where they will continue their PrEP care postrelease. Enrolled individuals are provided with an appointment date and time prior to their release, which is facilitated by a patient navigator who also helps to ensure the communication of important clinical information between the criminal justice clinical providers and the community site. The patient navigator also helps individuals address barriers that arise postrelease to facilitate retention in postrelease PrEP clinical care [56]. The primary clinical outcomes are the following: (1) PrEP uptake (yes/no prescription), (2) adherence (measured by self-report and dried blood spot), and (3) attendance at 1 follow up visit (yes/no attendance). The goals of this study are to increase our knowledge of the characteristics and HIV risk of criminal justice–involved men, improve our understanding of the PrEP care continuum in a criminal justice setting to address these risks, and evaluate real-world barriers to PrEP care among this vulnerable population. This study is expected to conclude on November 30th, 2022.

## Discussion

The results from this study will help to identify predictors for initiation, adherence, and retention in PrEP care among incarcerated and recently incarcerated men. This study will help characterize several key elements related to PrEP implementation that will need consideration in order for PrEP use to be implemented to scale in the criminal justice system in the United States. One important consideration for public health policy makers that work within the criminal justice system is the identification of individuals who meet clinical criteria for PrEP usage. Many individuals who pass through the criminal justice system are held for a brief period of time prior to sentencing (eg, pretrial). The study protocol presented here is designed to address gaps in the existing literature by documenting the number and type of individuals who meet the clinical criteria for PrEP, the organizational resources in the criminal justice setting, and the processes required to successfully implement PrEP care in this setting. For individuals who are detained for a significant period of time, particularly those who are sentenced to prolonged periods of detention, understanding how to incorporate HIV prevention into the criminal justice setting would be particularly helpful. Research on health-promoting interventions for HIV-negative criminal justice–involved individuals has generally focused on chronic care management during the period of transition from detention to community reentry [57]. This approach may benefit from incorporating HIV preventive care into the design of clinical linkage interventions.

Support from correctional leadership is key to the success of effective HIV prevention and PrEP programming. The commitment to effectively implement PrEP may vary significantly between different correctional departments, particularly given the cost of medications as well as the clinical and support personnel that might be required. In RIDOC, support from administrative and medical leadership has been key to the feasibility of conducting this study. Moving forward, a greater understanding of this variability and its impact on effective PrEP implementation will be critical. This will be particularly true if an injectable version of PrEP becomes available. A long-acting version of PrEP may provide greater protection from HIV acquisition, particularly in the chaotic postrelease period when adhering to a daily pill may be difficult for this population.

Beyond the consideration of adapting PrEP clinical processes to the criminal justice context, encouraging PrEP uptake for individuals from multiply marginalized backgrounds, linking individuals to care in the community postrelease, and addressing HIV risk and PrEP use in the context of the complex health needs of criminal justice–involved individuals are likely to require different approaches than may have been successful in other populations. Importantly, the criminal justice system represents an opportunity to reach and intervene with MSM who may not be traditionally reached otherwise (eg, LGBTQ+ engaged in health care). This is because criminal justice–involved men may be less likely to seek medical care in the community and more likely to have risk behaviors that are more highly stigmatized (eg, having sex with men for money, for drugs, or to meet other needs). There are likely to be some important differences among different populations experiencing incarceration who are eligible for PrEP, and characterizing these nuances in clinical care will be important to fully understanding what is required to encourage PrEP uptake and engagement in care among criminal justice–involved individuals. Finally, this population experiences many challenges in being linked to care while also experiencing extremely high risk for HIV acquisition [10,17,58,59]. Successful implementation of PrEP care within the criminal justice system and linking criminal justice–involved individuals to PrEP care in the community has the potential to significantly reduce the spread of HIV both within the criminal justice setting and the broader community, and is an important step to ending the HIV epidemic in the United States [60,61].

## Abbreviations

CDC: Centers for Disease Control and Prevention
MSM: men who have sex with men
PrEP: pre-exposure prophylaxis
RIDOC: Rhode Island Department of Corrections
TAF/FTC: tenofovir alafenamide and emtricitabine
TDF/FTC: tenofovir disoproxil fumarate and emtricitabine
